# Association between Frailty and Erectile Dysfunction among Chinese Elderly Men

**DOI:** 10.1155/2020/9247237

**Published:** 2020-07-07

**Authors:** Chengfu Li, Ji Sun, Huameng Zhao, Tingshan Dai

**Affiliations:** Department of Urology and Andrology, The Second Hospital of Fuyang People's Hospital, China

## Abstract

**Objective:**

This study is aimed at assessing association between frailty and erectile dysfunction among Chinese elderly men.

**Methods:**

This community-based study was conducted with a sample of 341 Chinese elderly men (aged 60 to 83 years old) in Fuyang City (Anhui Province, China). Each of the participants completed a standard questionnaire, including demographics (age, height, weight, yearly income, educational status, comorbidity, lifestyle factors, etc.), medical and sexual history, and the Chinese version of Tilburg Frailty Indicator (TFI) and International Index of Erectile Function-5 (IIEF-5) for assessing frailty and erectile dysfunction (ED).

**Results:**

The prevalence of ED and frailty in Chinese elderly men was 77.13% and 68.04%, respectively. Compared with the non-ED group, the ED group had increased age, spouse's age, BMI, prevalence of diabetes, and scores of TFI and lower yearly income, educational levels, and ratio of irregular intercourse (less than once per week) (all *P* < 0.05). Multivariate analysis indicated that age (OR: 0.860, 95% CI: 0.763-0.969), diabetes (OR: 0.330, 95% CI: 0.165-0.661), irregular intercourse (OR: 3.416, 95% CI: 1.874-6.229), and scores of TFI (OR: 0.906, 95% CI: 0.846-0.970) were regarded as independent risk factors for ED (all *P* < 0.05). And after adjusting for age, the TFI score had a negative significant association with the IIEF score (*r* = −0.134, *P* = 0.013).

**Conclusion:**

This study confirmed the strong associations between ED and frailty among elderly men. Sexual health care for elderly men with ED should be assessed and taken addressed on the multidimensional assessments of frailty.

## 1. Introduction

The aging of the global population is accelerating, and the percentage of people aged 60 and over is increasing every year. Currently, Japan and Europe have the highest percentage of population aged 60 and over. From 2009 to 2019, the percentage of aging people in China increased from 12.5% to 18.1% and will exceed 30% in 2050 [1]. The whole of society has faced great challenges taken by these demographic changes. Over the past few decades, we have made great success in improving the overall health and quality of life of elderly men [2–4]. However, as an important component of overall health, sexual health and sexual satisfaction have been seriously neglected, which can strongly decline the well-being of life among the aging population.

Erectile dysfunction (ED), the most common sexual dysfunction among men, tends to increase with advancing age [5, 6]. Studies report that 20–40% of men have had difficulty in achieving or maintaining erections in their 60s, and this increases to 50–70% for men aged 70 or over [7, 8]. In China, this percentage could be higher, as ED is not a life-threatening condition and elderly men may refuse to look for medical support [9]. Previous studies have shown that men with sexual problems were more likely to experience lower sexual satisfaction and quality of life [10, 11]. Given the issues reported above, it is essential to diagnose and treat ED. Owing to advances in basic research, we have taken deep insights into the pathology of ED, which revealed some risk factors affecting the potency of erections. Vascular health is regarded as the most important one [12]; others include nutrition, exercise, cardiovascular disease, obesity, diabetes, smoking, and lifestyle habits [13–15]. Additionally, we still face great challenges among elderly men, who have a more complex physiological and pathological status than young men. Importantly, aging, comorbidities, mental pressure, or combinations of these, as the special condition different from young men, have been proven to seriously affect erection [6, 14, 15].

Frailty is used to describe an age-related multidimensional dynamic physiological state with diminished resilience or vulnerability to stressors [16]. Frail elderly people often show a physiological decline in multiple organ systems, including the nervous, endocrine, circulatory, and immune systems, eventually leading to an increased risk of adverse health outcomes, such as falls, institutionalization, dementia, disability, and death [17]. In 2010, the Dutch scholar Gobbens proposed the integral conceptual model, which emphasized the dynamic development of physical, psychological, and social frailty. Subsequently, the Tilburg Frailty Indicator (TFI), based on the integral conceptual model, was created to provide a multidimensional assessment of frailty [18]. These adequate multidimensional frailty assessments were widely used in many countries and displayed great predictive validity and reliability [19, 20].

Despite both ED and frailty being connected with the aging process, there is little knowledge about the associations between frailty and ED, especially how frailty interrelates with erections among elderly men. Therefore, the purpose of this study is to explore the relationship between frailty and ED among elderly men. We also examine the independent risk factors for ED and expect to provide elderly men with better medical support for ED.

## 2. Materials and Methods

### 2.1. Study Population and Data Collection

This community-based cross-sectional study was performed in Fuyang City (Anhui Province, China) from July 2019 to October 2019. A total of consecutive 341 local men aged 60 or over were recruited in four communities who attended to community hospitals for health physical care. All subjects underwent routine examinations and assessment by a specific specialist on sexual medicine with five years of clinical experience, including sexual history and physical examination of the reproductive system. Subsequently, each participant who agreed to participate in this study would be guided by a specialized nurse into another consulting room to complete a written questionnaire, including the International Index of Erectile Function**-5** (IIEF-5), the TFI, medical and sexual histories, and conditions and demographic characteristics of spouses (such as age, height, weight, yearly income, educational status, comorbidity, lifestyle factors).

To be enrolled in this study, the participants must attempt sexual intercourse at least once per month with a stable partner for at least 6 months. Participants would be excluded if they suffered from serious psychiatric or somatic disorders or were addicted to any drug that could affect erectile function and/or psychological status. Men who had premature ejaculation or delayed ejaculation were also excluded.

This study was approved by the local ethics committee. All individuals who agreed to participate in this study signed written informed consent forms.

### 2.2. Frailty Assessment

Frailty, which is multidimensional, was assessed using the TFI questionnaire, which was divided into 3 domains and 15 items, including physical frailty (8 items), psychological frailty (4 items), and social frailty (3 items). Each item represented a score of 1, with the total score ranging from 0 to 15. A higher TFI score correlates with increasing levels of frailty. A total score > 5 was diagnosed as frailty [18].

### 2.3. ED Assessment

The IIEF-5, which has been widely used to evaluate whether elderly men experienced ED in the past 4 weeks, is composed of 5 questions related to ED. Participants choose a score of 0 to 5 for each question, with the total score of the IIEF being 0 to 25 and lower scores referring to more severe ED. A total score < 22 indicated the occurrence of ED [21].

### 2.4. Statistical Analysis

IBM SPSS Statistics 22.0 (SPSS Inc., Chicago, IL, USA) was used for statistical analysis. Quantitative data were expressed as mean ± SD, and the two-tailed unpaired Student's *t*-test was used to compare differences. Differences of proportions expressed as percentages were performed by the chi-squared test. Logistic regression analysis was used to estimate the odds ratios (ORs) and 95% confidence intervals (CIs) for the risk factors associated with ED. Linear correlation models were used to illuminate the association between the IIEF-5 and TFI scores. In order to eliminate the effect of age on the association, partial correlation was used to adjust for age. A *P* < 0.05 was considered statistically significant.

## 3. Results

### 3.1. Basic Characteristics of Participants and Comparison between Elderly Men with and without ED

This study enrolled 341 elderly men with a mean age of 70.26 ± 6.02 years, and the mean age of their spouses was 68.51 ± 5.98 years. According to the IIEF-5 and TFI, the prevalence of ED and frailty was 77.13% and 68.04%, respectively. The common comorbidities of participants were hypertension (58.7%) and diabetes (33.7%). In addition, the mean IIEF-5 and TFI scores were 13.27 ± 7.60 and 8.00 ± 4.44, respectively. Almost half of the participants had regular intercourse.

Statistical analyses suggested that the ED group had higher age and lower IIEF-5 score (age: 71.25 ± 5.83 vs. 66.92 ± 5.44, *P* < 0.001), indicating elderly men suffered from worse erections. It was also revealed that the ED group had higher mean age of the spouse and BMI and lower yearly income and educational level as compared to the non-ED group (all *P* < 0.05). No statistical differences were found between ED and residence, smoking, drinking, and healthy status of spouses (*P* > 0.05). In regard to the difference in the prevalence of comorbidities between the two groups, the results showed that the ED group was more likely to suffer from diabetes (*P* < 0.05), and no significant difference was found in the prevalence of hypertension (*P* > 0.05). Additionally, compared with the non-ED group, the ED group had a significantly higher TFI score (8.61 ± 4.29 vs. 5.95 ± 4.36, *P* < 0.001) and a higher ratio of regular intercourse (66.7% vs. 38.8%, *P* < 0.001). The detailed information is shown in Table 1.

### 3.2. Frailty Is an Independent Risk Factor for ED in Elderly Men

Bivariate logistic regression analysis was used to identify independent risk factors for ED. The results showed that age (OR: 0.860, 95% CI: 0.763 to 0.969), diabetes (OR: 0.330, 95% CI: 0.165 to 0.661), irregular intercourse (OR: 3.416, 95% CI: 1.874 to 6.229), and TFI score (OR: 0.906, 95% CI: 0.846 to 0.970) were regarded as independent risk factors for ED (all *P* < 0.05). The detailed information is presented in Table 2. Moreover, to illuminate the association between the IIEF-5 and TFI scores, a linear regression analysis was performed, indicating the TFI score had a significantly negative association with the IIEF-5 score (*r* = −0.296, *P* < 0.001). Considering the potential effect of age on the association, a partial correlation analysis was applied, and after adjusting for age, it was also found that the TFI score was negatively correlated with the IIEF-5 score (*r* = −0.134, *P* = 0.013).

## 4. Discussion

Currently, sexual health plays an important role in the overall health of elderly people. Because of the advanced aging process, the population of elderly people has risen rapidly. To improve their quality of life, great efforts have been made toward physical and mental health, while the sexual aspects have been ignored, with the most common sexual problem among elderly men being ED. Poor erections act importantly among elderly men with higher life expectancy.

Our study was conducted with a sample of 341 elderly Chinese men aged 60 to 83 years, with an ED prevalence of 77.13%. According to the Massachusetts Male Aging Study, 52% of men aged 40–70 years suffered from ED [22]. The Cologne Male Study reported an overall ED prevalence of 19.2% in men aged 30–80 years, with a steep age-related increase from 2.3% to 53.4% [23]. The differences in ED occurrence in these studies can be explained by differences in age, race, definition, methodology, and socioeconomic and cultural statuses of the study population.

Considering the higher prevalence of ED among elderly men, it is crucial to identify the risk factors for ED. Our study showed that the ED group had an increased age as compared with the non-ED group, and subsequent logistic regression analysis indicated age was regarded as an independent risk factor for ED. The studies by Ahn et al. and Yu et al. similarly confirmed the influence of increased age on ED, and each year of increase in age caused a decrease in the IIEF-5 score, meaning a more difficult erection condition [24, 25]. The decreases in coital frequency, physical activity, vascular function, and testosterone levels and increases in oxidative stress, inflammation, and glucose metabolic abnormalities with age played a vital role in the development of ED [26, 27]. Despite educational level and yearly income not being recognized as independent risk factors for ED, our study implied the ED group had lower educational level and yearly income. These could be attributed to higher educational level and income providing the elderly men with a better understanding of ED and effective medical support for ED treatment. Previous studies have highlighted a lack of knowledge and confidence for ED treatment in aged men. Moreover, they were afraid to involve doctors in their sexual problems or just neglected them because of the embarrassment and insufficient income, which were more common among elderly Chinese men [28–31]. Besides, our results showed that hypertension, smoking, and alcohol consumption were not found to be associated with ED, which was inconsistent with the results of previous studies [32–34]. This conclusion may be due to differences in methodology, sample scale, and socioeconomic and cultural factors. Given the comorbidities, the meta-analysis showed that diabetes was positively related to ED [15]. The observational results of our study were in line with the above. Good metabolic control acted importantly in vascular health, which was strongly associated with ED. Therefore, for aged men with diabetes, the control of blood sugar is essential. Interestingly, our study also found that having regular intercourse was important to decrease the occurrence of ED in elderly men. Similarly, a study by Koskimaki and colleagues also suggested that regular intercourse protected elderly men against the development of ED. The most likely explanation for this may be that the regular sexual activity protects vascular function and prevents cavernous fibrosis [35].

There are also some other factors associated with age that were also considered to be related to ED, such as the female partner's sexual dysfunction and mental disorders. Female sexual dysfunction is a common disease, which was associated with various demographic characteristics, including age. And it has been considered to be related to the male partner's erectile dysfunction. The research conducted by Nelson et al. in America found that there was a statistical correlation between female sexual function index and their partners' IIEF scores in 121 couples [36]. Even though the female partner's sexual function was not investigated in this study, it was also found that the partner's age in the ED group was higher compared with the group without ED. The increased age might be related to higher prevalence of female sexual dysfunction, which might be related to male erectile dysfunction. Additionally, psychological disorders were also regarded as risk factors of ED. Korfage et al. conducted a survey to examine the association between ED and mental health in healthy elderly men. And it was found that men with ED had poor mental health scores than subjects without ED [37].

Both frailty and ED could be recognized as a result of multisystem dysfunction with increasing age. However, there is little evidence on how frailty interrelates with ED. A study by Lee and colleagues suggested that frailty was associated with impaired overall sexual function, sex-related distress, and ED among older European men. In order to make deep insights into the association between frailty and ED, the multidimensional TFI was applied to detect frailty. Instead of the previous diagnostic tools, which only focused on physical frailty, the TFI emphasizes the multidimensional aspects of frailty and takes all three domains (physical, psychological, and social) into account. Several studies have stated that a multidimensional tool may be better to detect adverse outcomes [38–40]. Therefore, the prevalence of frailty in our study was 68.04% among elderly men, based on the total TFI scores. Compared with the non-ED group, the ED group showed high TFI scores, which was regarded as an independent risk for ED. Further linear regression analysis indicated the TFI scores had a significantly negative association with the IIEF-5 scores. Therefore, the TFI score may be of great predictive value for ED assessment. Importantly, sexual health care for ED should particularly pay more attention to the multidimensional insights of frailty, instead of focusing only on the physical aspect.

The study has several limitations including the following: firstly, this study was conducted in a single center with a tiny sample; secondly, difficulty in understanding the questionnaire (especially the IIEF-5 and TFI) may lead to the occurrence of information bias; thirdly, the evaluation of frailty in the sexual partner of elderly men was ignored; fourthly, sexual hormone was not tested for subjects in this study, and it may have an important effect on male erectile function. We will perform this test in the further study.

## 5. Conclusion

In conclusion, this cross-sectional study confirmed the strong associations between ED and frailty among elderly men, emphasizing the importance of a multidimensional assessment of frailty for elderly men suffering from ED. Medical support for ED should pay more attention to multidimensional aspects.

## Figures and Tables

**Table 1:** Demographics and clinical characteristics of elderly men with/without ED.

	Total subjects (*N* = 341)	With ED (*N* = 263)	Without ED (*N* = 78)	*t*/*χ*^2^	*P*
Age	70.26 ± 6.02	71.25 ± 5.83	66.92 ± 5.44	5.843	<0.001
BMI	24.13 ± 3.26	24.37 ± 3.31	23.35 ± 2.97	2.443	0.015
Residence				1.254	0.300
Urban	291 (85.4%)	143 (54.4%)	48 (61.5%)		
Rural	150 (14.6%)	120 (45.6%)	30 (38.5%)		
Income/year				5.211	0.033
>10,000 CNY	44 (12.9%)	28 (10.6%)	16 (20.5%)		
≤10,000 CNY	297 (87.1%)	235 (89.4%)	62 (79.5%)		
Education level				6.480	0.039
Primary school	248 (72.7%)	200 (76.0%)	48 (72.7%)		
Middle school	57 (16.7%)	38 (14.4%)	19 (16.7%)		
University	36 (10.6%)	25 (9.5%)	11 (10.6%)		
Smoking				0.567	0.518
Yes	157 (46.0%)	124 (47.1%)	33 (42.3%)		
No	184 (54.0%)	139 (52.9%)	45 (57.7%)		
Drinking				1.050	0.357
Yes	136 (39.9%)	101 (38.4%)	35 (44.9%)		
No	205 (60.1%)	162 (61.6%)	43 (55.1%)		
Diabetes				9.505	0.002
Yes	115 (33.7%)	100 (38.0%)	15 (19.2%)		
No	226 (66.3%)	163 (62.0%)	63 (80.8%)		
Hypertension				0.518	0.514
Yes	200 (58.7%)	157 (59.7%)	43 (55.1%)		
No	141 (41.3%)	106 (40.3%)	35 (44.9%)		
Regular intercourse				18.886	<0.001
Yes	154 (45.2%)	102 (38.8%)	52 (66.7%)		
No	187 (54.8%)	161 (61.2%)	26 (33.3%)		
Spouse age	68.51 ± 5.98	69.32 ± 5.78	65.81 ± 5.92	4.684	<0.001
Health status of spouse				0.937	0.626
Good	105 (30.8%)	84 (31.9%)	21 (26.9%)		
Fair	114 (33.4%)	88 (33.5%)	26 (33.3%)		
Poor	122 (35.8%)	91 (34.6%)	31 (39.7%)		
TFI	8.00 ± 4.44	8.61 ± 4.29	5.95 ± 4.36	4.798	<0.001
IIEF-5	13.27 ± 7.60	10.25 ± 5.87	23.47 ± 1.05	19.794	<0.001

^∗^Differences between elderly men with and without ED were assessed by *t*-test or chi-squared test, as appropriate. ED: erectile dysfunction; BMI: body mass index; CNY: Chinese yuan; TFI: Tilburg Frailty Indicator. Regular intercourse is defined as sexual intercourse ≥ once per week.

**Table 2:** Logistic regression analyses of potential risk factors for ED in elderly men.

	*β*	SE	Wals	*P* ^∗^	OR	95% CI for OR	
						Lower	Upper
Constant	9.617	2.519	14.576	<0.001			
Age	-0.151	0.061	6.132	0.013	0.860	0.763	0.969
BMI	-0.077	0.044	3.016	0.082	0.926	0.848	1.010
Income/year							
>10,000 CNY	-0.143	0.441	0.105	0.746	0.867	0.365	2.057
≤10,000 CNY					1.000		
Education							
Primary school					1.000		
Middle school	-0.139	0.465	0.089	0.766	0.871	0.350	2.167
University	-0.209	0.518	0.162	0.687	0.812	0.294	2.240
Diabetes							
Yes					1.000		
No	-1.109	0.355	9.782	0.002	0.330	0.165	0.661
Regular intercourse							
Yes					1.000		
No	1.229	0.306	16.074	<0.001	3.416	1.874	6.229
Spouse age	0.029	0.056	0.269	0.604	1.029	0.923	1.147
TFI	-0.098	0.035	7.944	0.005	0.906	0.846	0.970

^∗^Independent risks of ED were assessed by logistic regression analysis. ED: erectile dysfunction; BMI: body mass index; TFI: Tilburg Frailty Indicator.

## Data Availability

The datasets used and/or analyzed during the current study are available from the corresponding author on reasonable request.
