# Corrigendum to “Association between Frailty and Erectile Dysfunction among Chinese Elderly Men”

**DOI:** 10.1155/2021/5736092

**Published:** 2021-03-13

**Authors:** Chengfu Li, Ji Sun, Huameng Zhao, Tingshan Dai

**Affiliations:** Department of Urology and Andrology, The Second People's Hospital of Fuyang City, China

In the article titled “Association between Frailty and Erectile Dysfunction among Chinese Elderly Men” [1], all authors were affiliated to “Department of Urology and Andrology, The Second Hospital of Fuyang People's Hospital, China” which is incorrect.

The corrected affiliation is shown in the author information above.
